# Early kinesiophobia and its associated factors among patients after arthroscopic rotator cuff repair: a cross-sectional study based on latent profile analysis

**DOI:** 10.1186/s12891-025-09274-8

**Published:** 2025-11-27

**Authors:** Jingfang Cao, Guanzhong Yan, Yingjie Guo, Shengjuan Yan, Jiantao Guo

**Affiliations:** 1 School of Nursing, Inner Mongolia Medical University, Hohhot, Inner Mongolia Autonomous Region China; 2 The Second Affiliated Hospital of Inner Mongolia Medical University, No. 59, Horqin South Road, Saihan District, 010010, Hohhot, Inner Mongolia Autonomous Region China

**Keywords:** Postoperative rotator cuff repair patients, Kinesiophobia, Latent profile analysis, Influencing factors, Postoperative rehabilitation

## Abstract

**Background:**

Early postoperative kinesiophobia represents a significant psychological barrier in the rehabilitation trajectory following arthroscopic rotator cuff repair (ARCR). Existing research predominantly focuses on patients in the chronic phase, paying insufficient attention to the heterogeneity within this population. This study aimed to identify potential subtypes of kinesiophobia in patients after rotator cuff injury (RCI), analyze the causes affecting different subtypes of kinesiophobia, and provide a basis for formulating accurate early intervention plans.

**Methods:**

This study was conducted in the Department of Sports Medicine at the Second Affiliated Hospital of Inner Mongolia Medical University, China, from November 2024 to April 2025 (a duration of 6 months) using convenience sampling. This study included 245 consecutive participants who met the inclusion and exclusion criteria and who underwent arthroscopic ARCR after general anaesthesia. Finally, 237 participants completed this study. The Tampa Scale for Kinesiophobia(TSK,17 items, score range 17–68, ) was used as the primary outcome measure. The Self-Efficacy Scale(SEE; 9 items, 0–9 points), the “Changhai pain scale” Pain Score(0-10points), and the Groningen Orthopedic Social Support Scale (GO-SSS,12 items,0-36points) were used as secondary indicators. *Mplus8.0* was used for latent profiling analysis, and the Lo-Mendell-Rubin corrected likelihood ratio test (LMR-LRT, *p* < 0.05), Bayesian information criterion (BIC), and entropy value > 0.80 were used to ensure the reliability of classification, followed by univariate and multivariate logistic regression analyses of the classification results using *SPSS 25.0*.

**Results:**

237 patients were surveyed (51.10% male), with an average age of (58.08 ± 8.57) years. There were 89 (37.60%) patients with osteoporosis and 151 (63.70%) patients with full-thickness tears. These patients could be categorized into three latent profile groups: the low kinesiophobia-active group (17.70%), the moderate kinesiophobia-stable group (61.62%), and the high kinesiophobia-avoidance group (20.68%). Educational level（Junior high school and below: *p*=0.043, 95%CI 1.054–31.436); diabetes mellitus (*p*=0.033, 95%CI 1.111–12.364); exercise self-efficacy (*p*=0.022, 95%CI 0.720–0.975); osteoporosis (*p*=0.044, 95%CI 1.028–9.384); degree of tearing (*p*=0.015, 95%CI 1.317–13.631); pain level (*p*=0.002, 95%CI 1.543–7.217).

**Conclusion:**

Postoperative kinesiophobia in patients with rotator cuff injuries is at a medium to high level, with group heterogeneity. It is mainly influenced by biological factors (full-thickness tears, osteoporosis), clinical factors (history of revision surgery, pain level, chronic medical history), and psychosocial factors (low self-efficacy, low education). In the future, medical staff should identify patients with kinesiophobia as soon as possible, consider the multifaceted factors affecting this situation, and develop targeted interventions to reduce early postoperative kinesiophobia and enhance rehabilitation outcomes and improve quality of life.

**Supplementary Information:**

The online version contains supplementary material available at 10.1186/s12891-025-09274-8.

## Introduction

The rotator cuff is the shoulder joint’s most critical dynamic stabilising structure and includes the supraspinatus, infraspinatus, subscapularis, and teres minor muscles [1]. RCI remains a significant challenge in musculoskeletal medicine.RCI is the leading cause of shoulder dysfunction, and its incidence is increasing year by year [2, 3], with a prevalence of approximately 20% in the general population [4, 5] and up to more than 50% in people over 70 years of age [6]. The supraspinatus muscle is involved in the formation of the rotator cuff. It is the focal point of force in multiple directions of shoulder flexion and abduction [7], and therefore, the supraspinatus tendon is the most commonly damaged structure. For mild and moderate supraspinatus injuries (e.g., supraspinatus tendon abrasion, degeneration, inflammatory changes, etc.), conservative treatment can be used, but the rate of tendon-bone healing is low [8]. For severe supraspinatus tendon injuries (e.g., most of the supraspinatus tendon tear and complete rupture, etc.), and those who fail to be treated conservatively for 3 months, surgery can be used [9]. Currently, shoulder arthroscopy has become a mainstream modality for the treatment of rotator cuff injuries [10]. Early arthroscopic repair is recommended for active patients < 65 years, with 85% functional recovery rates when performed within 6 months of injury [11]. Even if the surgery is very successful, without effective rehabilitation, it may lead to joint stiffness and adhesion, pain, muscle atrophy, deep vein thrombosis, and even the risk of secondary surgery [12–15].

Kinesiophobia is a psychological state characterised by a fear of movement, in which an individual has an excessive fear of physical activity following trauma, which manifests itself in avoidance of exercise or even physical activity [16], and ultimately loss of function due to inactivity. A recent meta-analysis showed that patients after total knee arthroplasty have high levels of kinesiophobia early on [17].

Previous studies have shown that the majority of postoperative patients with RCI have high levels of kinesiophobia in the early stages [18], which hinders patients’ early rehabilitation and affects the recovery of shoulder function. Currently, there is a relative lack of research on kinesiophobia in patients with RCI, and the existing literature uses scale total scores for crude assessment [12, 19, 20], ignoring the heterogeneous characteristics of the patient population.

LPA is an individual-centred statistical analysis method that can be divided by potential profiles [21], which helps reveal heterogeneous subgroups of patients with kinesiophobia, elucidate the distribution and characteristics of each subtype. Therefore, this study aimed to (1) identify potential subtypes of kinesiophobia after RCI. (2) Determine factors associated with exercise phobia in individuals after RCI. By revealing the characteristics of the ‘subclinical fear’ group, the shortcomings of the traditional scale’s total score assessment will be remedied, and the development of individualised rehabilitation strategies will be promoted.

In this study, we explored the heterogeneity of postoperative kinesiophobia in RCI through LPA, which not only can make up for the shortcomings of the current research on the insufficient segmentation of patient groups, but also the results will directly guide the clinical practice: ① Provide researchers with a verifiable framework for the classification of subgroups, which will help the development of precision rehabilitation tools; ② Help clinicians to identify the high-risk patients through the predictive factors, and optimise the timing of the interventions for the follow-up of precision medical treatment; ③ Based on the Based on the results of our study, we can construct personalised rehabilitation programmes that suit the psychophysiological characteristics of patients, reducing their kinesiophobia and thus restoring their health.

We hypothesized that patients with kinesiophobia after RCI could be accurately divided into three subgroups using the LPA method, and the features between the groups were well distinguished. This study provides a reliable reference for clinical medical staff to intervene in cases of kinesiophobia.

## Methods

### Study design

This study was a cross-sectional survey conducted following the STROBE guidelines.

### Study setting and participants

This study was conducted in the Department of Sports Medicine at the Second Affiliated Hospital of Inner Mongolia Medical University, China, from November 2024 to April 2025 (6 months) using convenience sampling. The diagnosis of RCI in all patients was confirmed by our senior orthopaedic surgeons in the Department of Sports Medicine (deputy chief physician and above in the Second Affiliated Hospital of Inner Mongolia Medical University, who have been engaged in orthopaedic surgery for more than 10 years, and have completed 150 rotator cuff surgeries per year) based on the MRI manifestations and intraoperative arthroscopic explorations.

### Inclusion criteria

1.  Adults aged 18–70 years.
2. Patients with a partial or complete tear of the supraspinatus tendon of the rotator cuff after clinical examination and MRI require arthroscopic rotator cuff repair.
3. Meets the criteria for surgical treatment, including shoulder pain that significantly affects daily life or work, accompanied by limited shoulder joint movement, and conservative treatment is ineffective.
4. Informed consent and voluntary participation in this study.

### Exclusion criteria

1. Presence of severe cognitive dysfunction or mental disorder, unable to communicate.
2. Suffering from a severe disease or having suffered from any disease that impaired physical activity.
3. Patients with postoperative complications. (e.g., labral tear, cartilage damage).
4. Combination of other shoulder pathology (including acromioclavicular arthritis, osteoarthritis of the glenohumeral joint ≥ grade 2 or joint instability).
5. Presence of a large tendon tear (>5cm) or heavy fatty infiltration (Goutallier ≥3).
6. Need for other concurrent shoulder surgical interventions.
7. Professional athletes or occupations requiring heavy physical labour of the upper limbs.

### Sample size

**Fig. 1:**
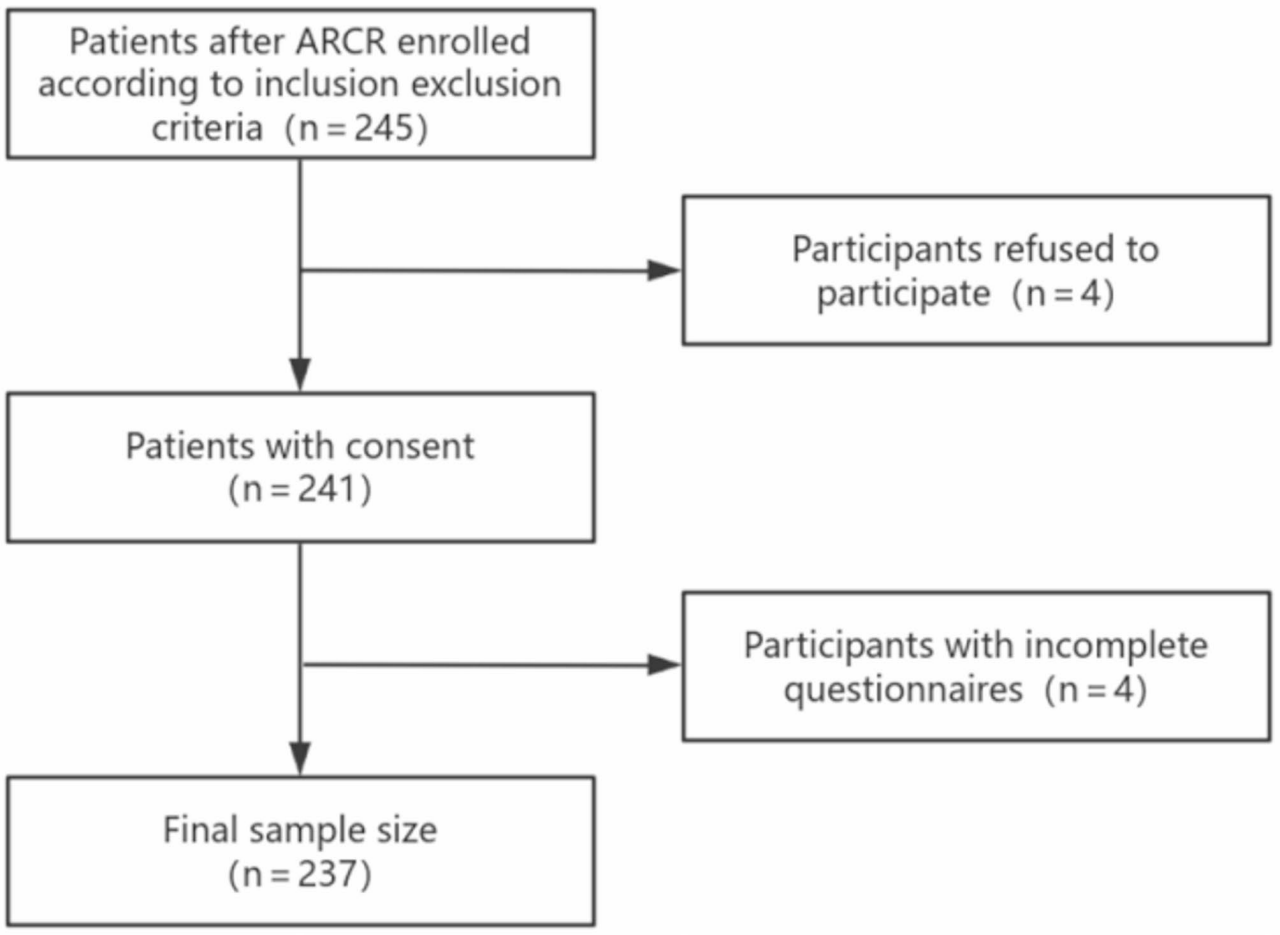
Participant recruitment flowchart

Based on Kendall’s multi-factorial research sample size method [22], the study included 18 independent variables, requiring a sample size of 5 to 10 times the number of independent variables. Considering a 20% dropout rate, a minimum of 228 completed questionnaires is required. Ultimately, a total of 237 questionnaires were distributed for this study (See Fig. 1).

### Instruments

#### Sociodemographic data and disease-related characteristics

A form was designed based on a literature review and consultation with the advice of sports medicine specialists, including gender, age, occupation, education, site of injury, degree of rotator cuff tear, and years of pain.

#### Tampa scale for kinesiophobia (TSK)

This questionnaire was developed by Tampa et al. to assess patients’ level of kinesiophobia. Hu [23] translated and modified this scale. The scale comprises seven dimensions and 17 items, each assigned 1–4 points, with a total score ranging from 17 to 68 points. Higher scores indicate a higher degree of kinesiophobia. The Cronbach’s alpha for the TSK scale was 0.778 in the original validation study [23], and in this study, it was 0.839.

#### Self-efficacy for exercise scale (SEE)

This questionnaire was developed by Resnick [24] et al. In this study, Lee’s translated and Chinese versions of the scale were used to measure the patient’s confidence in adhering to exercise in various situations. There were nine items, each with a score of 0–9. The higher the patient’s confidence in exercise, the higher the score. The Cronbach’s alpha for this SEE scale was 0.750 in the original validation study [25], and in this paper, 0.768.

#### Groningen orthopaedic social support scale (GO-SSS)

This questionnaire was developed by Dutch scholars Vanden [26] et al. and Chineseized by Sheng [27] et al. It was used to assess the social support of patients after RCI. The scale consists of perceived social support (7 items) and instrumental support (5 items). The scale was scored on a 4-point Likert scale from “never” to “often”, and the higher the score, the better the social support of the subject. The Cronbach’s alpha for the GO-SSS scale was 0.863 in the original validation study [27], and in this study it was 0.802.

#### The “Changhai Pain Scale” pain score

The “Changhai Pain Scale” [28] uses a scale of 0–10 to represent the degree of pain. The textual description of the pain sensation is easy for patients to understand and for healthcare personnel to assess. 0 means no pain, and 10 means severe pain. Combining the advantages of visual and numerical descriptions improves nursing education. User satisfaction was 88.28%, and patient satisfaction was 90%. The correlation coefficient between the two was 0.8241 [29].

### Data collection

Researchers trained in sports medicine explained the study protocol to all participants prior to enrollment, and written informed consent was obtained. The questionnaires were distributed on site in paper or electronic format with the participants’ informed consent and collected promptly. If there was any doubt, it was raised and resolved on site. Clinical cases were collected by manual screening. Invalid questionnaires were directly excluded.

### Data analysis

Data were independently entered by two researchers and cross-verified to ensure accuracy. *Mplus 8.0* and *SPSS 25.0* software were used for statistical analysis. This study utilized LPA to identify homogeneous subgroups of kinesiophobia based on TSK score patterns. This person-centered approach was chosen to capture the underlying heterogeneity within the population, moving beyond the use of arbitrary cut-off scores which may obscure meaningful subgroup differences First, LPA was performed using the 17 entries of the TSK to identify subgroups of motor fear in postoperative RCI patients. The Akaike Information Criterion (AIC), Bayesian Information Criterion(BIC), and adjusted Bayesian Information Criterion (aBIC) assessed model fit [30]. Lower AIC, BIC, and aBIC values imply better model fit. Lo-Mendell-Rubin (LMR) and Bootstrap Likelihood Ratio Test (BLRT) were used to test the model’s accuracy. If the *p*-values reached the significant level, it indicated that the k-category model was better than the k ~ 1-category model. For model classification precision, entropy values larger than 0.80 were considered appropriate. Latent class model characteristics were named based on each dimension’s mean line chart fluctuation.

*SPSS 25.0* was used for the descriptive statistics, univariate analysis, multicollinearity analysis, and regression analysis. Continuous variables were described using means and standard deviations, whereas categorical variables were summarized using frequencies and percentages. The potential fear category of RCI patients was used as the dependent variable for univariate analyses, and we used chi-square tests, analyses of variance (ANOVA), and Fisher’s exact probability to test for differences in population characteristics between the profiles. Variance inflation factor (VIF) was used to check for potential multicollinearity between variables of significance in univariate analyses, with VIF >5 indicating collinearity [31]. Comparisons between multiple groups were analysed using the Chi-square (χ^2^) test or Fisher’s exact probability method. Finally, multifactorial logistic regression was used to analyze the influence of kinesiophobia in RCI, and variables with *p* < 0.05 were discussed.

### Ethical considerations

This study was conducted in strict compliance with the ethical standards of the Declaration of Helsinki. Our study was reviewed by the Ethics Committee of the Second Affiliated Hospital of Inner Mongolia Medical University, China (approval number: EFY202500115). Written informed consent was obtained from all participants before data collection. Participants were fully informed about the purpose, procedures, potential risks, and benefits of the study, and their right to withdraw from the study at any time without consequence was emphasised. All data were treated confidentially according to ethical guidelines.

## Results

### Potential profile analysis

In this study, a total of 4 models were fitted using 17 entries from the kinesiophobia Scale as the exogenous variables, as shown in Table 1. When the number of potential profiles extracted was increased from 1 to 3, the AIC/BIC and aBIC all decreased. The LMR and BLRT tests were statistically significant (both *p* < 0.001). When the number of profiles increased from 3 to 4, neither the LRMT (*p* = 0.1149) nor the BLRT (*p* = 0.967) tests reached the level of significance. This suggests that the four-category profile model is inferior to the three-category profile model.

**Table 1:** Latent class model fit comparison

Model	AIC	BIC	aBIC	BLRT(*P*)	LMR(*P*)	Entropy	Probability of classes (%)
1	8017.087	8135.001	8027.233	-	-	-	-
2	7436.419	7616.758	7451.936	<0.001	<0.001	0.964	78.48/21.52
3	7118.689	7361.453	7139.578	<0.001	<0.001	0.954	17.70/61.62/20.68
4	6979.065	7284.254	7005.325	0.3437	0.3480	0.968	17.72/3.80/60.76/17.72

*AIC* Akaike Information Criterion, *BIC* Bayesian Information Criterion, *aBIC* adjusted Bayesian Information Criterion, *LMR (p)* P value for the Lo-Mendell-Rubin, *BLRT (p)* P value for the Bootstrapped Likelihood Ratio Test

In addition, from the matrix of attribution probabilities for the three categories of potential profile models (Table 2), the highest probability assigned to each category was for the third category (0.989), which further suggests that it is best to categorise the sport fear category into three categories.

**Table 2:** Three latent profiles of the probability matrices of patients’ kinesiophobia

Profile category	Class 1	Class 2	Class 3
Class 1	0.963	0.037	0.000
Class 2	0.007	0.986	0.007
Class 3	0.000	0.011	0.989

*C1 *Low Kinesiophobia-Activity Group, *C2 *Moderate Kinesiophobia-Stable Group, *C3 *High Kinesiophobia-Avoidance Group

**Fig. 2:**
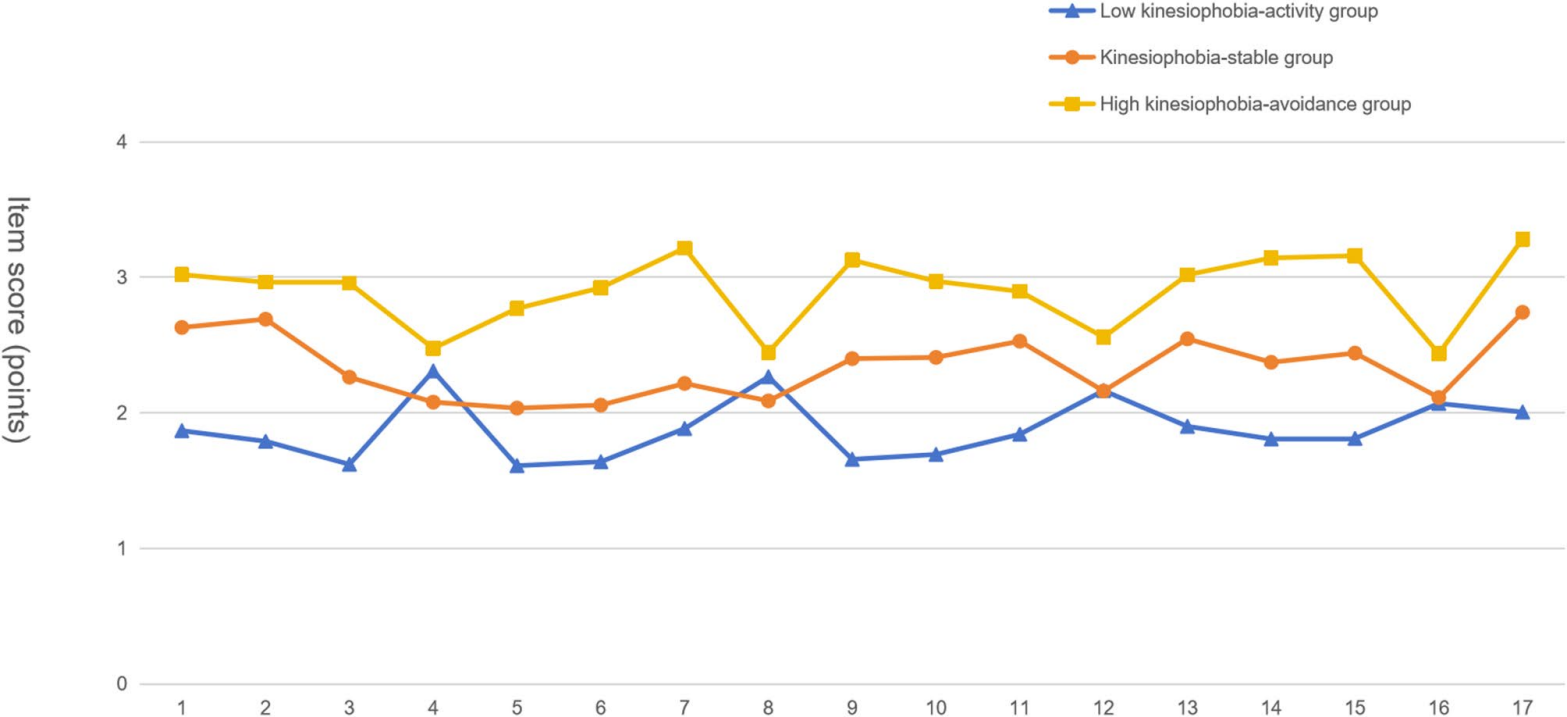
Item chart of kinesiophobia categories

Based on Fig. 2, the three latent profiles were categorized and named as follows: Class 1 (Low Kinesiophobia-Activity Group): Scores for this group were below two on most items except for items 4 (“If I exercise, my pain is likely to decrease”), 8 (“Just because an activity increases pain doesn’t mean it’s harmful”), and 12 (“I feel pain, but it would improve if I stayed active”). The scores exhibited minimal fluctuation, indicating that these patients had low kinesiophobia and believed early mobilization would facilitate recovery. Thus, this class was labeled the Low Kinesiophobia-Activity Group. Class 2 (Moderate Kinesiophobia-Stable Group): This was the largest subgroup, showing the least overall variation in scores, with a mean kinesiophobia score of 39.82 ± 2.017. Given the moderate and stable nature of their fear, this class was designated the Moderate Kinesiophobia-Stable Group. Class 3 (High Kinesiophobia-Avoidance Group): This group had the highest mean kinesiophobia score (49.43 ± 1.958), with item 17 (“I should not exercise when in pain”) scoring particularly high. This suggests a strong avoidance tendency due to pain-related fear, warranting the classification as the High Kinesiophobia-Avoidance Group.

### Characteristics of latent profile membership

Among the 237 postoperative RCI patients, statistically significant differences (*p* < 0.05) were observed across various demographic and clinical factors, including age, education level, presence of osteoporosis, comorbidities, and tear severity (Table 3).

**Table 3:** Participants’ demographic characteristics (*n* = 237)

Variables	Number(%)	Low Kinesiophobia - Active Group	Moderate Kinesiophobia - Stable Group	High Kinesiophobia - Avoidance Group	χ^2^ or F	*p*
Gender						
male	121(51.1)	23(54.8)	78(53.4)	20(40.8)	2.615	0.269^①^
female	116(48.9)	19(45.2)	68(46.6)	29(59.2)		
Age						
20–39	15(6.3)	7(16.7)	4(2.7)	4(8.2)	-	<0.001^②^
40–59	125(52.7)	24(57.1)	79(54.1)	22(44.9)		
60–79	97(40.9)	11(26.2)	63(43.2)	23(46.9)		
duration of pain						
<4 months	165(69.6)	33(78.6)	95(65.1)	37(75.5)	4.633	0.324^①^
4–8 months	47(19.8)	7(16.7)	31(21.2)	9(18.4)		
8–12 months	25(10.5)	2(4.8)	20(13.7)	3(6.1)		
ethnic group						
Han ethnic group	226(95.4)	41(97.6)	141(96.6)	44(89.8)	-	0.199^②^
Mongolian ethnic group	10(4.2)	1(2.4)	4(2.7)	5(10.2)		
Hui ethnic group	0(0.00)	0(0.0)	0(0.0)	0(0.0)		
other ethnic groups	1(0.4)	0(0.0)	1(0.7)	0(0.0)		
education level						
junior high school and below	89(37.6)	13(31.0)	45(30.8)	31(63.3)	17.745	<0.001^①^
high school or junior college	116(48.9)	19(45.2)	82(56.2)	15(30.6)		
college or bachelor’s degree and above	32(13.5)	10(23.8)	19(13.0)	3(13.5)		
place of residence						
city	151(63.7)	33(78.6)	96(65.8)	22(44.9)	11.766	0.003①
rural	86(36.3)	9(21.4)	50(34.2)	27(55.1)		
Occupation						
incumbency	74(31.2)	18(42.8)	39(26.7)	17(34.7)	5.331	0.257^①^
retired	81(34.2)	14(33.3)	53(36.3)	14(28.6)		
unemployed	82(34.6)	10(23.8)	54(37.0)	18(36.7)		
payment method						
medical insurance	214(90.3)	39(92.9)	133(91.1)	42(85.7)	1.595	0.450^①^
self-funded	23(9.7)	3(7.1)	13(8.9)	7(14.3)		
location of injury						
left shoulder	79(33.3)	10(23.8)	53(36.3)	16(32.7)	-	0.563^②^
right shoulder	157(66.2)	32(76.2)	92(63.0)	33(67.3)		
both shoulder	1(0.4)	0(0.0)	1(0.7)	0(0.0)		
diabetes mellitus						
exist	75(31.6)	7(16.7)	48(32.9)	20(40.8)	6.364^①^	0.042
none	162(68.4)	35(83.3)	98(67.1)	29(59.2)		
Osteoporosis						
exist	89(37.6)	9(21.4)	54(37.0)	26(53.1)	9.702	0.008
none	148(62.4)	33(78.6)	92(63.0)	23(46.9)		
degree of tearing						
partial tears	86(36.3)	17(40.5)	59(40.4)	10(20.4)	6.737	0.033
full-thickness tear	151(63.7)	25(59.5)	87(59.6)	39(79.6)		
surgical history						
exist	47(19.8)	5(11.9)	16(11.0)	26(53.1)	36.740	<0.001^①^
none	190(80.2)	37(88.1)	130(89.0)	23(46.9)		
SEE score		43.95±3.01	40.03±3.05	38.82±4.54	9.035	0.000^③^
GO-SSS score		26.86±2.84	27.72±2.11	27.253±2.53	7.853	0.001^③^
Pain score		1.83±0.79	2.33±0.72	3.37±0.70	8.409	0.000^③^
TSK score		31.93±2.908	39.82±2.017	49.43±1.958		

^①^ Chi-square test

^②^Fisher’s exact test

^③^Analysis of variance (ANOVA)

#### Multivariate analysis of kinesiophobia

The potential profile classification of kinesiophobia in patients after RCI was taken as the dependent variable (“low kinesiophobia-active group” as the reference), and the variables with statistical significance in the univariate analysis were used as independent variables; multiple logistic regression analysis was performed. Variables were coded in Table 4. The independent variable assignment method was shown in Table 5. Model fitting information showed χ2 = 105.700, *p* < 0.001, Cox & Snell R2 was 0.360, indicating good model fitting.

The logistic regression analysis revealed that educational level, chronic diseases, osteoporosis, tear severity, surgical history, pain level, and self-efficacy were key factors influencing the potential characteristics of kinesiophobia in postoperative RCI patients (see Table 5). The specific associations were as follows: individuals with a junior high school education or below had a significantly higher risk of being in the high kinesiophobia-avoidance group (C3) compared to the low kinesiophobia-active group (C1) (*p* = 0.043, 95% CI = 1.054–31.436)); patients with chronic diseases exhibited an increased risk in both the moderate kinesiophobia-stable group (C2) versus C1 (*p* = 0.040, 95% CI = 1.049–7.292) and C3 versus C1 (*p* = 0.033, 95% CI = 1.111–12.364); osteoporosis was associated with a significantly higher risk in C3 versus C1 (*p* = 0.044, 95% CI = 1.028–9.384); full-thickness tears were linked to an elevated risk in C3 versus C1 (*p* = 0.015, 95% CI = 1.317–13.631); a history of surgery significantly increased the risk in C3 versus C1 (*p* = 0.001, 95% CI = 2.471–30.833); higher pain levels were correlated with an increased risk in the C3 group (*p* = 0.002, 95% CI = 1.543–7.217); and lower self-efficacy was associated with a higher risk in the C3 group (*p* = 0.022, 95% CI = 0.720–0.975).

**Table 4:** Assignment of independent variables

variables	Assignment method
education level	Junior high school and below, high school or junior college=2, College or bachelor’s degree or above=3;
diabetes mellitus	Yes=1, no=2;
osteoporosis	Yes=1, no=2;
degree of tearing	Partial tears=1, Full-thickness tear=2;
pain score	Original value input
SEE score	Original value input
GO-SSS score	Original value input
surgical history	Original value input

**Table 5:** Multivariate analysis of kinesiophobia

Variables		C2 vs.C1				C3 vs.C1				tolerance	VIF
		B	*p*	OR	95%CI	B	*p*	OR	95%CI		
constants		1.405	0.705	-	-	2.698	0.551	-	-	-	-
education level	Junior high school and below	0.243	0.666	1.276	0.422,3.857	1.750	0.043	5.757	1.054,31.436	0.940	1.064
	High school or junior college	0.228	0.597	1.334	0.472,3.700	0.261	0.764	1.229	0.236,7.151		
diabetes mellitus	yes	1.017	0.040	2.766	1.049,7.292	1.310	0.033	3.706	1.111,12.364	0.934	1.071
osteoporosis	yes	0.607	0.171	1.835	0.770,4.373	1.113	0.044	3.106	1.028,9.384	0.952	1.050
degree of tearing	Full-thickness tear	0.078	0.847	1.081	0.490,2.387	1.444	0.015	4.237	1.317,13.631	0.959	1.042
surgical history	Yes	−0.217	0.715	0.805	0.251,2.584	2.167	0.001	8.728	2.471,30.833	0.947	1.055
SEE score		−0.117	0.048	0.889	0.792,0.999	−0.177	0.022	0.838	0.720,0.975	0.918	1.089
GO-SSS score		0.158	0.055	1.171	0.996,1.376	−0.100	0.359	0.905	0.730,1.121	0.942	1.062
pain score		1.003	0.001	2.727	1.524,4.880	1.205	0.002	3.337	1.543,7.217	0.949	1.054

*C1 *Low Kinesiophobia-Activity Group, *C2 *Moderate Kinesiophobia-Stable Group, *C3* High Kinesiophobia-Avoidance Group, *VIF* Variance inflated factor, *B *Coefficient (Beta), *PP*-value, *OR *Odds Ratio, *95%CI *95% Confidence Interval

## Discussions

### Heterogeneity in kinesiophobia levels among postoperative RCI patients

This study identified three distinct latent classes of kinesiophobia during the early postoperative period using LPA, demonstrating significant heterogeneity in kinesiophobia among this population. The mean kinesiophobia score was 40.41 ± 5.877. ①Low Kinesiophobia-Activity Group (17.70%): Exhibited minimal kinesiophobia and higher activity engagement. Most believed early mobilization could alleviate pain, reflecting proactive coping strategies.②Moderate kinesiophobia - stable group accounted for 61.62% of the patients in this category, with a moderate level of kinesiophobia, which is the primary type of kinesiophobia in rotator cuff injuries. According to the kinesiophobia score, most of the patients in this group still believe that they should not exercise when they are in pain, although they have the support of external systems. This suggests that most of the postoperative patients with rotator cuff injuries have symptoms of kinesiophobia in the early stages. It suggests that active implementation of physical and psychological interventions in this group could further reduce kinesiophobia and improve prognosis.③High Kinesiophobia-Avoidance Group(20.68%): Scored highest across all items, particularly Item 17 (“No one should exercise when in pain”). Demonstrated maladaptive pain-movement associations (“activity-pain-reinjury” cycle), likely due to: Poor health literacy, Catastrophic misinterpretation of minor movements. Clinical implication: It requires early identification and multimodal interventions (such as health education and graded exposure therapy) to break fear-avoidance patterns [18].

### Analysis of factors influencing the potential profile categories of kinesiophobia in postoperative patients with rotator cuff injuries

The results of this study indicate that an educational level of junior high school or below was identified as a risk factor for classification in the high kinesiophobia-avoidance group. This finding is consistent with the report by Kararti et al. [19]. A possible explanation may be that patients with lower educational attainment generally possess relatively limited health literacy, which can significantly hinder their ability to comprehend and acquire rehabilitation-related knowledge [32]. This impairment may lead to misconceptions about rehabilitation, heightening concerns about re-injury, and consequently reinforcing avoidance behavior. Given these observations, greater clinical attention should be directed toward patients with low educational levels. It is recommended that healthcare providers prioritize health education initiatives tailored to this population, utilizing simplified materials such as illustrations, short videos, and interactive lectures to improve disease-related knowledge. Such approaches may help correct misconceptions and ultimately reduce kinesiophobia.

Patients with osteoporosis were found to be more likely to belong to the high kinesiophobia-avoidance group. Osteoporosis is characterized by reduced bone mass and a significant increase in bone fragility [33], heightening fear of exercise-induced fractures. These patients face greater risks of skeletal weakness and susceptibility to injury [34]. One possible explanation is that lower bone mineral density leads to concerns that upper limb activity may provoke a proximal humerus fracture following RCI repair, prompting early avoidance of movement. Additionally, patients with osteoporosis often experience a fear of falling. As a rotator cuff injury impairs upper limb function, this further exacerbates kinesiophobia. Therefore, healthcare providers should emphasize “safe movement” rather than “activity avoidance,” and collaborate with multidisciplinary teams to develop integrated rehabilitation plans that address both functional recovery of the shoulder and bone protection. Incorporating anti-osteoporotic therapy into the rehabilitation regimen may help alleviate patients’ concerns about movement-related risks, enhance exercise confidence, and ultimately reduce kinesiophobia.

The results of this study indicate that patients with full-thickness tears exhibit higher levels of movement-related fear, consistent with Liu’s findings [35], which suggests that the greater the tear severity, the more pronounced the patients’ kinesiophobia-related risks. However, this finding contrasts with Zhang’s conclusion that “more minor tears cause more significant pain [36]. ” This discrepancy may reflect characteristic manifestations at different stages of the disease process: acute-phase pain in patients with more minor tears is primarily associated with neural sensitization and inflammatory responses. In comparison, chronic-phase kinesiophobia in patients with larger tears is more attributable to the long-term psychological impact of joint instability and functional loss [37]. Additionally, patients who have already undergone arthroscopic surgery and those who have not yet decided on surgical treatment exhibit similarly low levels of disease awareness [38], believing that greater tear severity correlates with higher pain levels and stronger kinesiophobia. This finding suggests that clinical interventions should be tailored based on the severity of the tear. For patients with small tears, the focus should be on anti-inflammatory pain relief. In contrast, for those with large tears, psychological intervention should be combined with health education to help them understand that post-surgical pain is not entirely due to their subjective perceptions, correcting misconceptions, and assisting patients in reducing post-surgical rehabilitation movement phobia levels to promote the recovery of shoulder joint function.

Patients with diabetes mellitus (DM) were found to have a higher probability of being classified into the high kinesiophobia-avoidance group following rotator cuff injury (RCI). This is consistent with previous national and international studies identifying diabetes as a risk factor for RCI [39, 40]. A potential explanation may be that the majority of RCI patients are elderly, and those with comorbid diabetes often experience impaired balance due to peripheral neuropathy, which can further amplify fear of movement. Additionally, some patients may avoid exercise training due to concerns that physical activity could induce hypoglycemia [41]. However, an alternative perspective suggests that patients with diabetes may develop stronger health awareness and self-regulatory skills through long-term disease management, potentially facilitating greater engagement in rehabilitation [42, 43]. The present findings support the former view, indicating that although proactive self-management is possible, this population remains more susceptible to fear-avoidance patterns overall. These results underscore the need for clinicians to actively address the psychological concerns of postoperative RCI patients with diabetes and develop individualized rehabilitation plans. Such tailored interventions should mitigate kinesiophobia, support functional recovery, and improve overall quality of life.

Early functional rehabilitation is an essential component of postoperative care following shoulder arthroscopy. However, joint pain and swelling after surgery may reduce patients’ willingness to engage in physical activity. Persistent pain often leads to fear of rehabilitation exercises, resulting in avoidance of functional training [44]. The findings of this study demonstrate that increased pain levels act as a driving factor in the development and progression of kinesiophobia, which is consistent with the results reported by Suer et al. [45]. As a potent negative stimulus, pain has been shown to amplify the fear of movement [46] significantly. Nevertheless, pain is considered a modifiable factor. Through targeted interventions, kinesiophobia can be improved, pain symptoms effectively alleviated, and the process of functional recovery accelerated [18]. It has been suggested that effective physical therapy interventions may mitigate the negative impact of kinesiophobia on rehabilitation, thereby reducing pain, enhancing muscle strength, and lowering the degree of kinesiophobia [47]. Therefore, healthcare providers are advised to assess patients’ pain levels at an early stage and implement effective physical and psychological interventions—such as multimodal preemptive analgesia [48], cognitive-behavioral therapy, and pain neuroscience education—to help improve shoulder pain and subsequently reduce kinesiophobia.

Self-efficacy is an individual’s judgment and belief in their ability to perform in specific situations. Patients with higher levels of self-efficacy often assess pain more objectively during the post-surgical rehabilitation process and demonstrate stronger determination when implementing rehabilitation training [49]. The results indicate that exercise self-efficacy negatively influences the latent classes of kinesiophobia, which is consistent with the findings reported by Butera et al. [50]. This may be attributed to the fact that patients with lower self-efficacy tend to reduce their engagement in rehabilitation exercises due to concerns about performing movements incorrectly and a lack of confidence in recovery, ultimately leading to avoidance behavior. In contrast, individuals with higher self-efficacy are more likely to confront and overcome their fears, actively participating in physical activities. In contrast, those with lower self-efficacy often adopt passive coping strategies and consciously avoid opportunities for movement. These insights highlight the need for healthcare professionals to identify patients with low exercise self-efficacy, encourage them to express their thoughts and emotions, and strengthen their confidence in rehabilitation. Enhancing patients’ exercise self-efficacy can facilitate adopting lifelong regular physical activity, reducing their kinesiophobia.

Furthermore, we identified a history of previous surgery as one of the factors predicting the development of high levels of kinesiophobia. This is consistent with recent studies [51, 52], which suggest that unmet expectations from the initial surgery or unsuccessful rehabilitation—often leading to revision surgery—can serve as a strong negative feedback, significantly exacerbating psychological trauma and fear of movement. The underlying mechanism may lie in the fact that secondary surgery not only signifies the “failure” of the initial treatment and prolongs the patient’s “sick role,” but may also involve more complex pathology, a longer recovery process, and more pessimistic expectations regarding functional restoration [53]. Such experiences can reinforce the patient’s perception of bodily vulnerability and the mistaken belief that movement may cause re-injury, leading to long-term avoidance behaviors that persist even after tissue healing. For these patients, clinical management must go beyond conventional physical therapy. Comprehensive psychosocial assessment should be prioritized, along with early integration of Pain Neuroscience Education (PNE) and Cognitive Behavioral Therapy (CBT) to address catastrophic thinking. Graded exposure therapy in a safe and controlled setting can also help rebuild confidence in movement.

### Clinical implications and subgroup-specific recommendations

This study utilized LPA to reveal that kinesiophobia in patients after RCI is not a homogeneous phenomenon. This finding may encourage healthcare professionals to shift from generalized intervention models toward precise and individualized management strategies, enabling accurate patient stratification and targeted interventions. To guide the future clinical translation of these findings, we propose a structured framework aimed at informing the development of personalized intervention strategies for different profiles (Table 6).

**Table 6:** Proposed targeted intervention strategies based on kinesiophobia profiles

Profile Group	Defining Characteristics	Primary Intervention Goals	Recommended Strategies
Low Kinesiophobia-Active	Low fear, low pain, high self-efficacy, higher education.	Maintain motivation, prevent overactivity.	Provide advanced exercise prescription; encourage peer leadership; educate on signs of overuse.
Moderate Kinesiophobia-Stable	Moderate fear, sta-ble but hold mis-conceptions.	Cognitive restructuring, build confidence.	Structured pain education (PNE); sup-ervised function-al training; goal sett-ing with gradual progression.
High Kinesiophobia-Avoidant	High fear, catastro-phic thinking, av-oidance.	Reduce fear, break avoidance cycle, build trust.	Multimodal approach: CBT + GradedExposure Therapy; prioritize pain and c-omorbidity management; strong ps-ychological support.

Further detailed clinical recommendations can be found in the supplementary material [see Additional file 1]

### Limitations

Although this study preliminarily identified latent classes of kinesiophobia and their influencing factors in patients after rotator cuff repair, several limitations should be acknowledged. First, the most critical restriction stems from the cross-sectional design. Data on kinesiophobia and potential influencing factors were collected simultaneously at a single postoperative time. This “snapshot” approach inherently precludes causal inferences between variables. Second, the study did not comprehensively measure or control for all possible confounding factors, which may have introduced bias. Examples include postoperative medication use, specific pain characteristics, and postoperative complications(such as labral tears or cartilage damage). The absence of these variables may have altered the actual effects observed. Finally, all scales used in this study were self-reported instruments. Although validated, they are still susceptible to recall bias and reporting bias. Patients may have underreported their level of fear due to social desirability or provided pain ratings influenced by their immediate emotional state. In conclusion, future research should employ prospective longitudinal cohort designs to clarify causal relationships and incorporate more comprehensive and detailed data on medication, pain characteristics, and behavioral outcomes. Such approaches would help build more robust predictive models and provide more substantial evidence for developing targeted clinical interventions.

## Conclusion

In summary, early kinesiophobia in patients following rotator cuff repair can be classified into three distinct categories: a low kinesiophobia-active group, a moderate kinesiophobia-stable group, and a high kinesiophobia-avoidence group. Multiple factors, including educational level, osteoporosis, tear severity, diabetes, self-efficacy, pain intensity, and surgical history, influence this classification. In clinical practice, greater emphasis should be placed on the early assessment and identification of postoperative psychological states. Implementing multidimensional and individualized intervention strategies will help alleviate kinesiophobia, enhance shoulder functional recovery, and improve overall surgical outcomes and patient quality of life.

## Supplementary Information

Supplementary Material 1.

## Data Availability

The data used to support the funding of this study are available from Jiantao Guo upon request.

## Abbreviations

ARCR: Arthroscopic rotator cuff repair
RCI: rotator cuff injury
LPA: Latent profile analysis
TSK: Tampa Scale for Kinesiophobia
ANOVA: One-way analysis of variance
LMR: Lo-mendell-rubin
BLRT: Bootstrap likelihood ratio test
AIC: Akaike information criterion
BIC: Bayesian information criterion
