# Supplementary material for: Early kinesiophobia and its associated factors among patients after arthroscopic rotator cuff repair: a cross-sectional study based on latent profile analysis
Source: BMC Musculoskelet Disord. 2025 Nov 27;26:1076. doi: 10.1186/s12891-025-09274-8 (PMC12659394; doi:10.1186/s12891-025-09274-8)
Supplement: Supplementary file 1 — Supplementary Material 1. [file 12891_2025_9274_MOESM1_ESM.docx]

| **Profile Group** | **Defining Characteristics & Key Risk Factors** | **Primary Intervention Goals** | **Specific, Actionable Intervention Strategies** |
| --- | --- | --- | --- |
| Low Kinesiophobia-Active | 1. Psychological: Low TSK scores. Positive beliefs about exercise benefits. High exercise self-efficacy.  2. Clinical: Lower pain levels. Lower prevalence of osteoporosis and surgical history.  3. Social: Higher education level. | 1. Maintain high motivation and exercise adherence.  2. Prevent overactivity and re-injury risk.  3. Leverage as a positive peer influence. | 1. Maintaining Physical Positivity: Encourage the patient to sustain their current level of rehabilitation activities. Provide positive feedback to reinforce their constructive beliefs.  2. Differentiated Pain Education: Educate the patient on distinguishing between "benign pain" (e.g., muscle fatigue, stretching sensation) and "adverse pain" (e.g., sharp pain, catching/locking sensation, or radiating pain). Guide them to adjust activities based on the nature of the pain rather than its mere presence.  3. Peer Motivation: Encourage their participation as a "model patient" in peer support education, while cautioning against the risk of early overactivity due to overconfidence. Alternatively, suggest they share their positive experiences in patient education seminars. |
| Moderate Kinesiophobia-Stable | 1.Psychological: Moderate TSK score. Endorses the misconception "I should not exercise when in pain."  2.Clinical: Moderate pain levels. May have comorbidities like diabetes . Mix of partial and full-thickness tears. | 1. Correct maladaptive beliefs about the relationship between pain and movement. 2. Build confidence through guided, successful movement experiences. 3. Prevent regression into avoidance patterns. | Pain Neuroscience Education (PNE): Utilize metaphors and diagrams to explain central sensitization and how post-operative pain can become a "false alarm." Target and correct the specific maladaptive belief that "pain equals harm."  2. Supervised, Success-Oriented Movement: Begin with therapist-guided passive/active-assisted range of motion exercises. Employ phrasing such as, "Let's see if we can safely move your arm to 45 degrees," to reframe the goal from "pain-free" movement to "safe" movement.  3. Cognitive Reappraisal: Implement a simple thought record: "What goes through your mind when you feel this pain? Is there another, more helpful way to understand this sensation?" |
| High Kinesiophobia-Avoidant | 1.Psychological : High TSK scores. Strong catastrophic thinking (e.g., high agreement with "I should not exercise when in pain"). Low exercise self-efficacy.  2.Clinical: High pain intensity. High prevalence of key risk factors: osteoporosis, full-thickness tears, previous surgical history, and diabetes .  3.Social: Lower education level. | 1. Break the maladaptive fear-avoidance cycle.  2. Build trust in the repaired shoulder and the rehabilitation process. 3. Manage high pain and address comorbidities to create a foundation for rehab. | **1.Cognitive Behavioral Therapy (CBT):** Directly target catastrophic thinking and fear-avoidance beliefs. Regular communication between the therapist and the rehabilitation team is essential.  **2.Graded Exposure Therapy (GET):**  **Co-creation of a Fear Hierarchy:** Collaboratively develop a list of feared activities, ranked from the least anxiety-provoking (e.g., imagining moving the arm) to the most (e.g., raising the hand overhead).  **Systematic Exposure:** Begin at the lowest level of the hierarchy. The patient repeatedly performs the activity until their subjective fear (SUDS rating) significantly decreases.  3.**Multimodal Analgesia:** Coordinate with physicians to ensure effective pain management (e.g., scheduled NSAIDs, cryotherapy) prior to rehabilitation sessions to lower the initial barrier to participation.  **4.Comorbidity Proactive Management:** Close collaboration with physicians is crucial. Optimize management of osteoporosis (e.g., calcium, vitamin D, pharmacotherapy) and diabetes (stabilize blood glucose) to reduce the patient's perceived biological risk of movement. |

**Table 6. Proposed Targeted Intervention Strategies Based on Kinesiophobia Profiles**
